# Role of Human Organic Cation Transporter 1 (hOCT1) Polymorphisms in Lamivudine (3TC) Uptake and Drug-Drug Interactions

**DOI:** 10.3389/fphar.2016.00175

**Published:** 2016-06-24

**Authors:** Cristina Arimany-Nardi, Gerard Minuesa, Thorsten Keller, Itziar Erkizia, Hermann Koepsell, Javier Martinez-Picado, Marçal Pastor-Anglada

**Affiliations:** ^1^Molecular Pharmacology and Experimental Therapeutics, Department of Biochemistry and Molecular Biology, Institute of Biomedicine, University of BarcelonaBarcelona, Spain; ^2^Oncology Program, National Biomedical Research Institute on Liver and Gastrointestinal Diseases (CIBER EHD), Instituto de Salud Carlos IIIMadrid, Spain; ^3^AIDS Research Institute IrsiCaixa, Institut d'Investigació en Cièncias de la Salut Germans Trias i Pujol, Universitat Autònoma de BarcelonaBadalona, Spain; ^4^Department of Pharmacology, School of Medicine, University of WürzburgWürzburg, Germany; ^5^Department of Molecular Plant Physiology and Biophysics, Julius-von-Sachs-Institute, University of WürzburgWürzburg, Germany; ^6^Universitat de Vic - Universitat Central de CatalunyaVic, Spain; ^7^Institució Catalana de Recerca i Estudis Avançats (ICREA)Barcelona, Spain; ^8^Institut de Recerca Pediàtrica Hospital Sant Joan de DéuBarcelona, Spain

**Keywords:** hOCT1, pharmacogenetics, lamivudine, HIV infection, therapy

## Abstract

Lamivudine (3TC), a drug used in the treatment of HIV infection, needs to cross the plasma membrane to exert its therapeutic action. Human Organic cation transporter 1 (hOCT1), encoded by the *SLC22A1* gene, is the transporter responsible for its uptake into target cells. As *SLC22A1* is a highly polymorphic gene, the aim of this study was to determine how SNPs in the OCT1-encoding gene affected 3TC internalization and its interaction with other co-administered drugs. HEK293 cells stably transfected with either the wild type form or the polymorphic variants of hOCT1 were used to perform kinetic and drug-drug interaction studies. Protein co-immunoprecipitation was used to assess the impact of selected polymorphic cysteines on the oligomerization of the transporter. Results showed that 3TC transport efficiency was reduced in all polymorphic variants tested (R61C, C88R, S189L, M420del, and G465R). This was not caused by lack of oligomerization in case of variants located at the transporter extracellular loop (R61C and C88R). Drug-drug interaction measurements showed that co-administered drugs [abacavir (ABC), zidovudine (AZT), emtricitabine (FTC), tenofovir diproxil fumarate (TDF), efavirenz (EFV) and raltegravir (RAL)], differently inhibited 3TC uptake depending upon the polymorphic variant analyzed. These data highlight the need for accurate analysis of drug transporter polymorphic variants of clinical relevance, because polymorphisms can impact on substrate (3TC) translocation but even more importantly they can differentially affect drug-drug interactions at the transporter level.

## Introduction

The human Organic Cation Transporter 1 (hOCT1), encoded by the *SLC22A1* gene, is a plasma membrane transporter protein responsible for the translocation of a broad range of drugs currently used, among others, in anti-diabetic, antiemetic, antihypertensive, anticancer and antiviral therapies (Koepsell et al., 2007; Nies et al., 2011; Arimany-Nardi et al., 2015a). hOCT1 is expressed in major epithelial barriers thereby being a suitable candidate to determine drug bioavailability and action (Tzvetkov et al., 2009; Nies et al., 2011). The synthesis of fully active hOCT1 proteins requires the formation of homo-oligomers. Cysteines located at the extracellular loop between transmembrane domains (TMD) 1 and 2 are known to be implicated in the oligomerization of hOCT1 by forming di-sulphide bonds (Keller et al., 2011).

Moreover, *SLC22A1* is a highly polymorphic gene with many of its polymorphic variants present in a high percentage of the population (Kerb et al., 2002; Shu et al., 2003; Arimany-Nardi et al., 2015a). Some of these polymorphic variants lead to amino acid substitutions and, at least in one case, can even induce deletions, as for the M420del variant. In most cases these hOCT1 polymorphic variants have been shown to significantly alter the capacity of the transporter to translocate its substrates in a manner which can differentially affect substrate specificity. In fact, a paradigm in the field of drug transporter pharmacogenetics is the cellular handling of metformin by hOCT1, its polymorphic variants being responsible for a variety of pharmacokinetics and pharmacodynamics effects (Ahlin et al., 2011).

The antiviral drug lamivudine (3TC) has also been shown to be a suitable hOCT1 substrate (Minuesa et al., 2009). Interestingly, two rare variants found in Asian population have been shown to alter 3TC uptake by hOCT1 (Jung et al., 2008; Choi and Song, 2012). 3TC is a broadly used drug for the treatment of Human Immunodeficiency Virus (HIV) and Hepatitis B virus (HBV) infections. High doses of the drug are used in monotherapy for the treatment of HBV infection, while low drug doses are used in combination with other antiretroviral agents (mostly, abacavir -ABC- and azidothymidine -AZT-) for the treatment of HIV infection (Johnson et al., 1999). Moreover, several of these antiretroviral drugs, which are being used in combination for the treatment of HIV infection, have been also shown to interact with hOCT proteins (Jung et al., 2008; Minuesa et al., 2009). However, this interaction might be mechanistically complex as they can bind with high affinity to the transporter allosterically inhibiting substrate translocation, without being transported themselves. Essentially, high affinity binding of other antiviral nucleosides might result in inhibition of 3TC uptake, thereby anticipating the occurrence of drug-drug interactions at the transporter level in AIDS therapy (Minuesa et al., 2009).

hOCT1 is highly expressed in hepatocytes, being a good candidate to play a major role in the efficacy of 3TC treatment of HBV infection (Nies et al., 2009). Interestingly enough, hOCT expression has also been detected in immune cells. Both hOCT1 and hOCT3 have been shown to be expressed in monocytes, Monocyte-Derived Macrophages (MDMs) and Monocyte-Derived Dendritic Cells (MDDCs) and also in Peripheral Blood Mononuclear Cells (PBMCs) and CD4+ T cells (Minuesa et al., 2008). In fact, hOCT1 together with hOCT2 have also been reported to be responsible for the accumulation of 3TC in CD4+ cells of HIV-infected patients (Jung et al., 2013). Moreover, hOCT1 mRNA expression has been shown to be increased in the lymph nodes of HIV-infected patients in comparison to healthy controls (Jung et al., 2008).

How hOCT1 genetic heterogeneity might affect 3TC-transporter interaction and, more importantly, how hOCT1 polymorphisms might determine drug-drug interactions at the transporter level, has not been studied so far. In this study, we have undertaken the analysis of the ability of hOCT1 polymorphic variants to interact and translocate 3TC, and how this interaction can be affected by the presence of other antiretroviral drugs currently used in the clinics. Considering that two of the described hOCT1 polymorphic variants (hOCT1R61C and hOCT1C88R) implicate cysteine residues, which are known to be located at the extracellular loop between TMDs 1 and 2, the impact of these two variants on the ability of the hOCT1 protein to oligomerize has also been assessed in order to unveil any structural constraints associated with these genetic variants.

## Materials and methods

### Reagents

N-Methyl-4-phenyl pyridinium iodide (MPP^+^) and lamivudine (3TC) were obtained from Sigma-Aldrich (St. Louis, MO). Abacavir (ABC), zidovudine (AZT), emtricitabine (FTC), tenofovir diproxil fumarate (TDF), efavirenz (EFV) and raltegravir (RAL) were obtained from the NIH AIDS Research and Reference Reagent Program. [Methyl-^3^H]-N-Methyl-4-phenyl pyridinium iodide ([^3^H]MPP^+^) was obtained from American Radiolabeled Chemicals, Inc. (St. Louis, MO), [5-3H(N)]lamivudine ([^3^H]3TC) was purchased at, Hartmann Analytic GmbH (Braunschweig, Germany).

### Molecular biology

The polymorphic substitutions in hOCT1 (GenBank accession number X98322) (Gorboulev et al., 1997) were introduced using the primers shown in Table 1. The constructs generated were verified by DNA sequencing (BigDye Terminator v3.1, Applied Biosystems, Foster City, CA).

**Table 1:** **Primers (5′-3′) used to introduce the polymorphic variations into hOCT1**.

R61C	Fw	CTGGGGTGGCTGAGCTGAGCCAGTGCTGTGGCTGGAGCCCTGCGGAGG
	Rv	CCTCCGCAGGGCTCCAGCCACAGCACTGGCTCAGCTCAGCCACCCCAG
C88R	Fw	GGGCGAGGCCTTCCTTGGCCAGCGCAGGCGCTATGAAGTGGACTGG
	Rv	CCAGTCCACTTCATAGCGCCTGCGCTGGCCAAGGAAGGCCTCGCCC
F160L	Fw	GTCCTGTTTGAATGCGGGCTTCCTCTTTGGCTCTCTCGGTGTTGGC
	Rv	GCCAACACCGAGAGAGCCAAAGAGGAAGCCCGCATTCAAACAGGAC
S189L	Fw	GAACTGTGCTGGTCAACGCGGTGTTGGGCGTGCTCATGGCCTTCTCGCC
	Rv	GGCGAGAAGGCCATGAGCACGCCCAACACCGCGTTGACCAGCACAGTTC
P341L	Fw	CATTTGCAGACCTGTTCCGCACGCTGCGCCTGAGGAAGCGCACCTTC
	Rv	GAAGGTGCGCTTCCTCAGGCGCAGCGTGCGGAACAGGTCTGCAAATG
G401S	Fw	CCCTCATCACCATTGACCGCGTGAGCCGCATCTACCCCATGGCCATGTC
	Rv	GACATGGCCATGGGGTAGATGCGGCTCACGCGGTCAATGGTGATGAGGG
M408V	Fw	GGGCCGCATCTACCCCATGGCCGTGTCAAATTTGTTGGCGGGGGCAG
	Rv	CTGCCCCCGCCAACAAATTTGACACGGCCATGGGGTAGATGCGGCCC
M420Del	Fw	GGCGGGGGCAGCCTGCCTCGTCATTTTTATCTCACCTGACCTGC
	Rv	GCAGGTCAGGTGAGATAAAAATGACGAGGCAGGCTGCCCCCGCC
G465R	Fw	CCCCACATTCGTCAGGAACCTCCGAGTGATGGTGTGTTCCTCCCTG
	Rv	CAGGGAGGAACACACCATCACTCGGAGGTTCCTGACGAATGTGGGG

*The variations introduced have been underlined in forward (Fw) and reverse (Rv) primers*.

For generating the stable expressing cell lines, hOCT1, hOCT1R61C, hOCT1C88R, hOCT1S189L, hOCT1M420Del or hOCT1G465R were recloned into the pcDNA5/FRT/TO vector (Invitrogen, Carlsbad, CA). Restriction sites were added using the following primers (restriction sites underlined): 5′- CGGGGTACCATCATGCCCACCGTGGATGACA -3′ and 5′- CGCGGATCCCTCTCAGGTGCCCGAGGGTTC -3′ and the amplification product was subcloned into KpnI and BamHI sites of pcDNA5/FRT/TO vector.

### Cell culture

Human embryonic kidney 293 cells (HEK293) were routinely maintained at 37°C/5% CO_2_ in Dulbecco's modified Eagle's medium (Lonza Verviers SPRL, Verviers, Belgium) supplemented with 10% heat-inactivated fetal bovine serum (vol/vol), 2 mM glutamine, and a mixture of antibiotics (100 U penicillin, 0.1 mg/ml streptomycin, and 0.25 mg/ml fungizone) (Life Technologies, Paisley, UK).

FlpIn-HEK293 cells were maintained in the same medium supplemented with 100 μg/ml hygromycin B (Life Technologies, Paisley, UK).

### Transfection of hOCTs in HEK293 cells and cell culture

HEK293 cells were transiently transfected using calcium phosphate.

To generate the stable cell lines the eukaryotic expression vectors and an empty vector (pcDNA5), used as a control in uptake experiments, were transfected into the FlpIn-HEK293 cell line (Life Technologies, Paisley, UK) using calcium-phosphate and selected for positive clones with 100 μg/ml hygromycin B (Life Technologies, Paisley, UK).

Cell clones with the highest transport activity were chosen for further study. They were routinely cultured in Dulbecco's modified Eagle's medium (DMEM) (Lonza Verviers SPRL, Verviers, Belgium) supplemented with 10% heat-inactivated fetal bovine serum (v/v), 2 mM glutamine, and a mixture of antibiotics (100 U penicillin, 0.1 mg/ml streptomycin, and 0.25 mg/ml fungizone) (Life Technologies, Paisley, UK) in the presence of 100 μg/ml hygromycin B. They were maintained at 37°C in a humidified atmosphere containing 5% CO_2_.

### Uptake measurements

[^3^H]MPP^+^ and [^3^H]3TC uptake in HEK293-hOCTs and/or HEK293-pcDNA5 (empty vector) cells was measured after 1 s (MPP+) and 15 s (3TC) incubations as previously described (Minuesa et al., 2009).

For kinetics studies increasing concentrations of the non-labeled drug were used and the obtained values were fitted into a Michaelis-Menten curve to calculate the *Km* and *Vmax* values.

### Co-precipitation of his-tagged and FLAG-tagged transporters

Target proteins were cell free expressed as previously reported (Keller et al., 2011). The precipitates of the target proteins were then solubilized in Tris buffer containing 2% 1-myristoyl-2-hydroxy-snglycero-3-[phospho-rac-(1-glycerol)](LMPG), followed by centrifugation for 10 min at 13,000 g. To investigate protein-protein interactions LMPG supernatant (100 μL with 20 μg of protein) containing one *in vitro*-expressed transporter with a His tag was mixed with LMPG supernatant (100 μL with 20 μg of protein) containing an *in vitro*-expressed FLAG-tagged transporter and diluted with 200 μL of Tris buffer containing 1% CHAPS and 10 mM imidazole (final detergent concentrations, 1% LMPG and 0.5% CHAPS) and incubated for 1 h at 4°C; 100 μL of Ni2+-NTA-agarose equilibrated with Tris buffer containing 1% LMPG, 0.5% CHAPS, and 10 mM imidazole was added and the suspension incubated for an additional 1 h at 4°C, and the beads were separated via centrifugation for 10 min at 500 g (room temperature). The beads were washed five times at room temperature with 1 mL of Tris buffer containing 1% CHAPS and 10 mM imidazole-CHAPS and five times with Tris buffer containing 1% CHAPS and 20 mM imidazole. For protein elution, pelleted beads were suspended for 10 min at room temperature in 400 μL of Tris buffer containing 1% CHAPS and 100 mM imidazole, and the suspension was centrifuged for 10 min at 500 g. The supernatants were collected, and FLAG-tagged transporters were analyzed by Western blots as previously described (Keller et al., 2011).

### Statistical analysis

Paired and unpaired *t*-Student test were carried out using GraphPad Prism 4.0 software for the statistical comparison of experimental data.

## Results

### Effect of hOCT1 polymorphisms in lamivudine, metformin and MPP^+^ uptake

The impact of hOCT1 polymorphic variants on the uptake of 3TC was assessed. For this purpose, the most common polymorphic variants of the *SLC22A1* gene were generated by site-directed mutagenesis of the reference (wild type) cDNA template and transiently transfected with these vectors in HeLa cells. The uptake rates of 10 μM [^3^H]3TC and 1 μM [^3^H]MPP^+^ by these transporter variants were measured and compared to the ones for metformin published by Shu et al. (2007) (Supplementary Figure 1). Transport activities expressed as the percentages of total wild type-associated transport are shown in Table 2.

**Table 2:** **hOCT1 studied SNPs defined by rs number and amino acid (AA) change**.

**dbSNP**	**AA change**	**Allele Freq Caucasians (%)^1^**	**MPP+ uptake 1 min**	**3TC uptake 1 min**	**Metformin uptake 5 min^3^**
rs12208357	R61C	0.7	75	40	5
rs55918055	C88R	0.62^2^	10	25	n.d
rs683369	F160L	0.65	100	100	100
rs34104736	S189L	0.5	80	50	25
rs2282143	P341L	0	100	85	100
rs34130495	G401S	1.1	10	25	10
rs628031	M408V	59.8	100	100	100
rs35167514	M420del	18.5	80	50	30
rs34059508	G465R	4	10	20	5

*The different compounds uptake is expressed as percentage of hOCT1 wild type uptake. MPP^+^ and 3TC uptake measurements were performed in transiently transfected HeLa cells. Those polymorphic variants highlighted in gray were chosen for being further characterized and stable HEK293 cells expressing them were generated*.

1 *(Shu et al., 2003)*.

2 *(Kerb et al., 2002)*.

3 *(Shu et al., 2007)*.

Interestingly, polymorphic variants did not similarly affect the uptake of all drugs, being the most striking differences in substrate selectivity those found in variants R61C, S189L, and M420del. Those polymorphisms combining both a relatively high allelic frequency in humans, and substrate-dependent altered transport function (variants R61C, C88R, S189L, M420del, and G465R; highlighted in Table 2) as well as the wild type hOCT1 were chosen to generate a panel of HEK293 cell lines, stably expressing each single polymorphic hOCT1 protein. This panel of cell lines is indeed useful for drug screening purposes.

### Kinetic parameters of MPP^+^ and 3TC uptake mediated by hOCT1 SNPs

**Figure 1:**
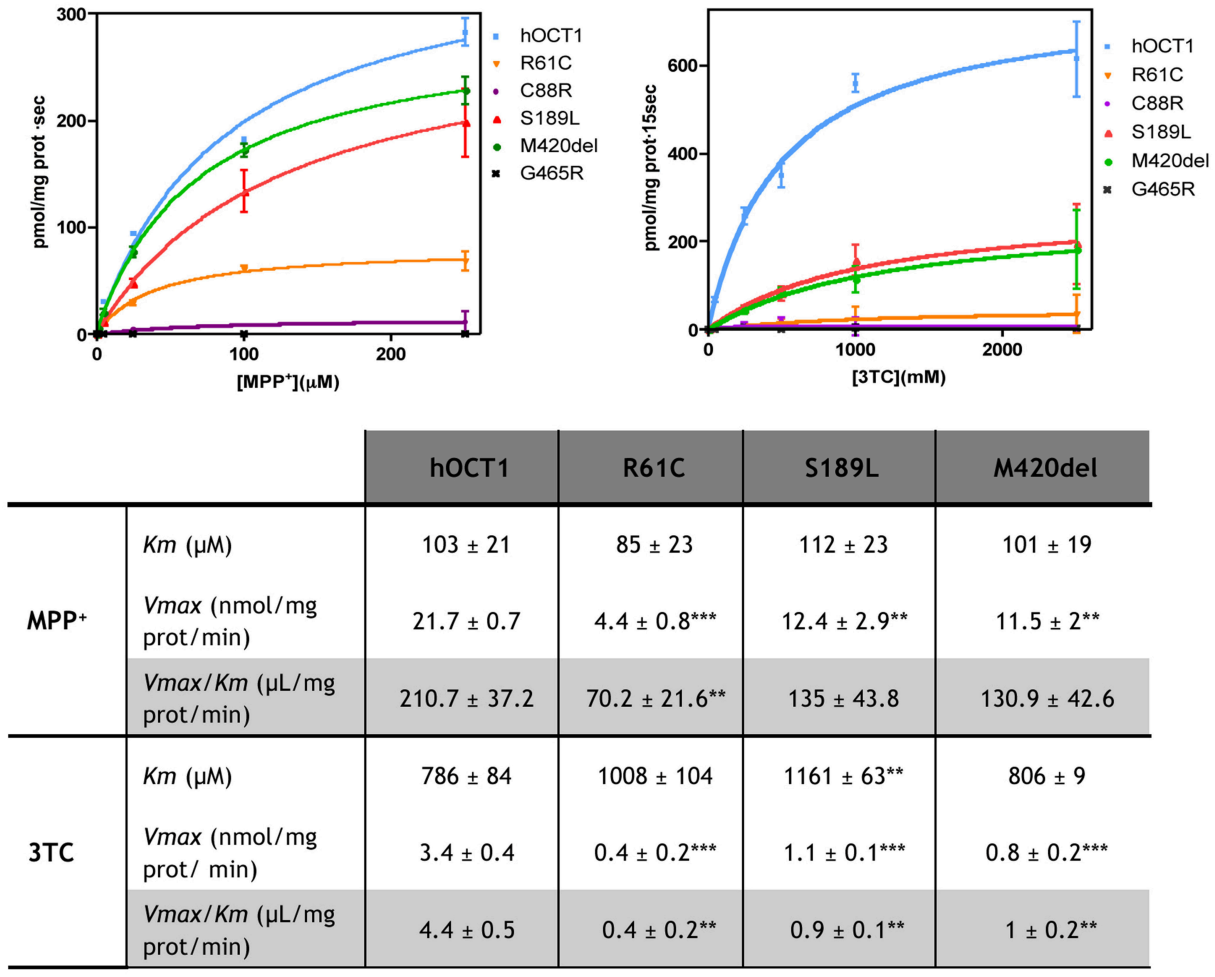
**Kinetic studies for 3TC and MPP^**+**^**. Kinetic studies were performed in HEK293 stably expressing hOCT1 wild type or one of the polymorphic variants. For MPP^+^ the uptake was measured at 1 s and for 3TC at 15 s to enhance the sensibility. Kinetic parameters shown in the table were calculated fitting data to a Michaelis Menten curve. Unpaired *t*-Student test was performed. (^**^p < 0.01; ^***^p < 0.001).

HEK293 cells stably expressing the polymorphic variants R61C, C88R, S189L, M420del, and G465R were used for the characterization of MPP^+^ and 3TC uptake kinetics. These determinations were performed at 1 s for MPP^+^ and at 15 s for 3TC. Under these conditions, non-hOCT1-related MPP+ uptake is negligible (Supplementary Figure 2). Results are shown in Figure 1.

*Km* and *Vmax* values were calculated by fitting the experimental data to a *Michaelis Menten* curve. In case of the C88R and G465R variants, data could not be fitted because uptake rates for both substrates mediated by these variants were too low. *Km* values for MPP^+^ were similar to the wild type transporter for all tested variants. However, for 3TC, the S189L *Km* value was significantly higher (*p* < 0.01) than for the wild type transporter. Although *Vmax* values for all drugs were significantly decreased in all polymorphic variants, they were particularly much lower when 3TC was the assayed substrate than when using the hOCT1 substrate model MPP^+^, thereby anticipating differential interaction properties in a substrate-dependent manner. Transport efficiency was calculated by dividing *Vmax* by *Km* values. Ratios above 1 were considered to be associated with adequate transporter efficacy. Nevertheless, measurements were performed on cell clones stably overexpressing these transporter variants, which contributes to increase transport efficacy. In any case, based upon this premise, it can be concluded that even though MPP^+^ transport efficiency was significantly reduced for the R61C transporter (3.4-fold, *p* < 0.01) this variant could still be considered an effective transporter if expressed at levels high enough as to warrant efficient substrate translocation. In fact, it seems that none of the assayed hOCT1 polymorphic transporters showed a significant impairment in MPP^+^ uptake, despite a general tendency to decreased efficacy, being transport efficiency values very high in all tested cell lines. This was completely different when the same panel of transporter variants were assayed for 3TC transportability. Transport efficiency for 3TC was comparatively much lower than for the hOCT1 model substrate MPP+, albeit still being consistent with hOCT1 being a suitable 3TC transporter as previously determined (Minuesa et al., 2009). However, in this case, all tested polymorphic hOCT1 proteins showed a dramatic impairment in transport efficiency when compared to the wild type transporter, being the *Vmax/Km* ratio close to or even lower than 1 (showing a 11-fold decrease for R61C and >4-fold for S189L and M420del, all *p* < 0.01).

### Oligomerization of hOCT1 and two of its polymorphic variants located at the large extracellular loop

**Figure 2:**
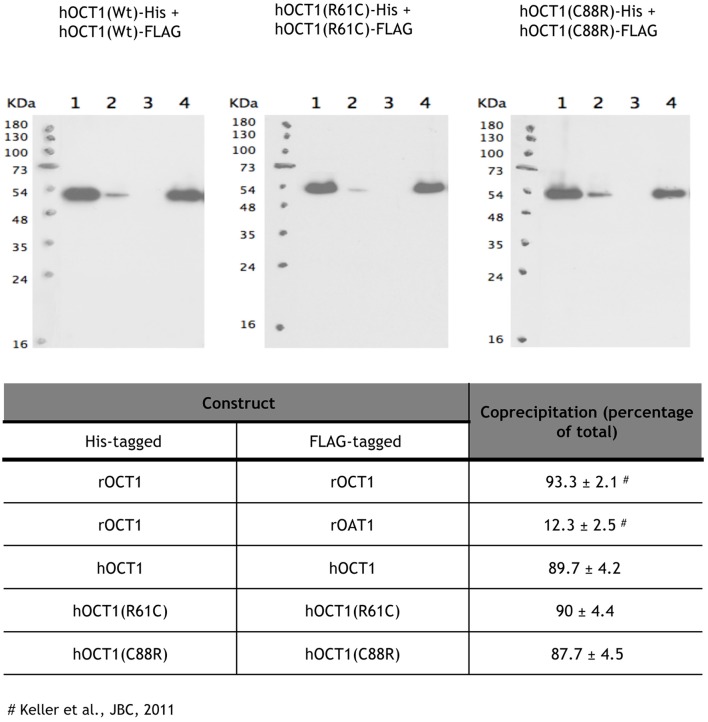
**Oligomerization of polymorphic variants located at the extracellular loop**. The samples were diluted with 200 μL of Tris buffer containing 1% CHAPs and 10 mM imidazole (lanes 1). After 1 h incubation at 4°C, Ni^2+^-NTA-agarose beads were added, the suspension was incubated for 1 h and centrifuged. Supernatants were collected (lanes 2). The beads were washed five times with 1 mL of buffer and pelleted (supernatants lanes 3). His-tagged proteins were eluted by incubating the beads with 400 μl of buffer containing 1% CHAPS and 100 mM imidazole (supernatant lanes 4). Proteins were separated by SDS-PAGE, transferred to a blotting membrane, and stained with an antibody against the FLAG tag. The experiment was performed three independent times. The mean ± S.D. are shown in the table.

Two of the studied polymorphic variants implicate cysteines (R61C and C88R) located at the large extracellular loop of the hOCT1 protein. They either add a new cysteine (R61C) within the loop or substitute an already existing one by arginine (C88R). Cysteines located at the extracellular loop of human Organic Cation Transporters have recently been implicated in the oligomerization and proper trafficking to the plasma membrane of hOCT2 (Brast et al., 2012). Oligomerization studies were performed to determine if these polymorphic variants affecting cysteines at the extracellular loop could still form oligomers. For this purpose, His-tagged and FLAG-tagged hOCT1 WT and the polymorphic variants of interest were incubated with Ni^2+^-NTA-agarose beads able to bind His-tagged proteins, as previously reported for rOCT1 (Keller et al., 2011). FLAG-tagged proteins bound to His-tagged proteins in all cases, except for the FLAG-rOAT1 which could not oligomerize with His-rOCT1. This was used as a negative control of the assay (Figure 2). Data were consistent with the view that both R61C and C88R variants were still able to oligomerize, although it does not necessarily mean that oligomers are inserted into the plasma membrane as efficiently as for the wild type proteins. These observations also suggest that the cysteine at the 88 position is not essential for the correct oligomerization of the transporter.

### Drug-drug interactions in hOCT1 polymorphic variants

Management of HIV-1 infection usually includes the combination of multiple antiretroviral drugs. Besides 3TC, other Nucleoside Reverse Transcriptase Inhibitors (NRTI) antiviral drugs, such as ABC, have been shown to interact with hOCT1, despite not being translocated by the transporter (Minuesa et al., 2009). In addition, in the case of metformin, the polymorphic variant M420del has been determined to be more sensitive to drug-drug interactions than the wild type transporter (Ahlin et al., 2011). These facts raised the question of what would be the pharmacological impact of these variants on drug-drug interactions of clinical relevance.

**Figure 3:**
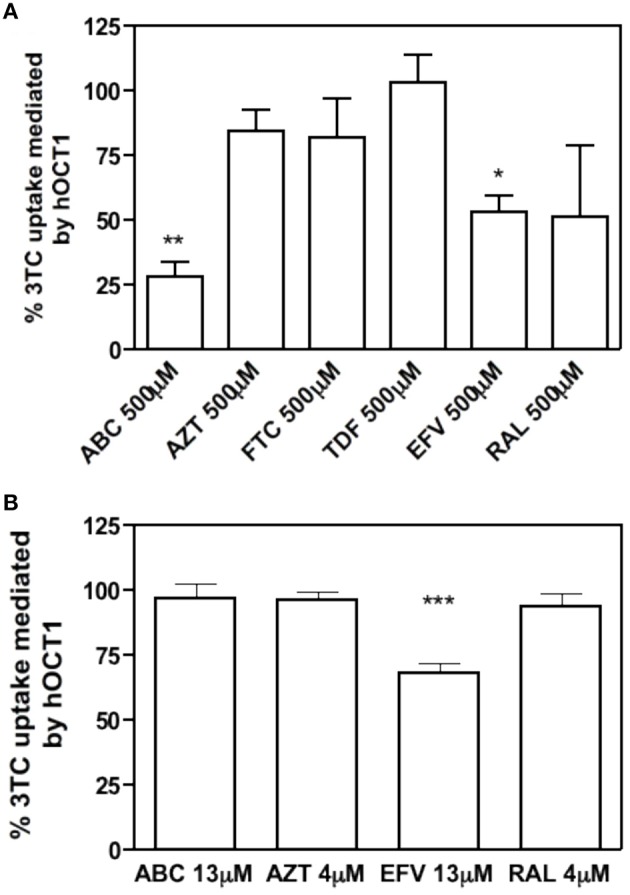
**3TC uptake inhibited by other antiretroviral drugs**. Percentage of inhibition of 500 μM antiretroviral drugs (up) or *Cmax* concentration (down) to the uptake of 10 μM, 2 μCi of 3TC mediated by hOCT1. The uptake was measured at 15 s. The graph shows the mean ± S.E.M of three independent experiments. (^*^p < 0.05; ^**^p < 0.01; ^***^p < 0.001).

To address this issue, different antiretroviral drugs which are often co-administrated with 3TC were selected. The tested drugs were ABC, AZT, FTC, and TDF from the Nucleoside Reverse Transcriptase Inhibitors (NRTIs) group; EFV from the Non-Nucleoside Reverse Transcriptase Inhibitor (NNRTI) group and RAL form the HIV-1 Integrase Inhibitors group. A fixed high concentration (500 μM) for each interacting drug and 10 μM, 2 μCi of [^3^H]3TC as substrate were initially used to perform a *cis-inhibition* profiling of the wild type hOCT1 transporter, stably expressed in HEK293 cells, as mentioned above. We were aware that this concentrations was far beyond the reported maximum concentrations (*Cmax*) for all these drugs, but we designed this study simply as a preliminary step to identify putative interacting candidates. Results shown in Figure 3, demonstrate that ABC, EFV, and RAL inhibited hOCT1-mediated 3TC uptake. These drugs were selected for further studies along with AZT, one of the drugs which is more often co-administrated with 3TC. The fact that AZT could not inhibit the wild type hOCT1 does not rule out the possibility of this drug showing altered interactions with the polymorphic transporters. *Cmax* representative concentrations (Table 3, Jilek et al., 2012) were also used for further *cis*-inhibition studies. Under those conditions, only EFV could significantly inhibit 3TC uptake mediated by the wild type transporter (Figure 3).

**Table 3:** ***Cmax (nature med 2012* Jilek) and concentration used of the antiretroviral drugs for the cis-inhibition studies**.

**Drug**	**Class**	***Cmax* (μM)**	**Used concentration (μM)**
3TC	NRTI	15, 29	10
ABC	NRTI	13, 48	13
AZT	NRTI	4, 45	4
EFV	NNRTI	12, 97	13
RAL	InST1	4, 50	4

**Figure 4:**
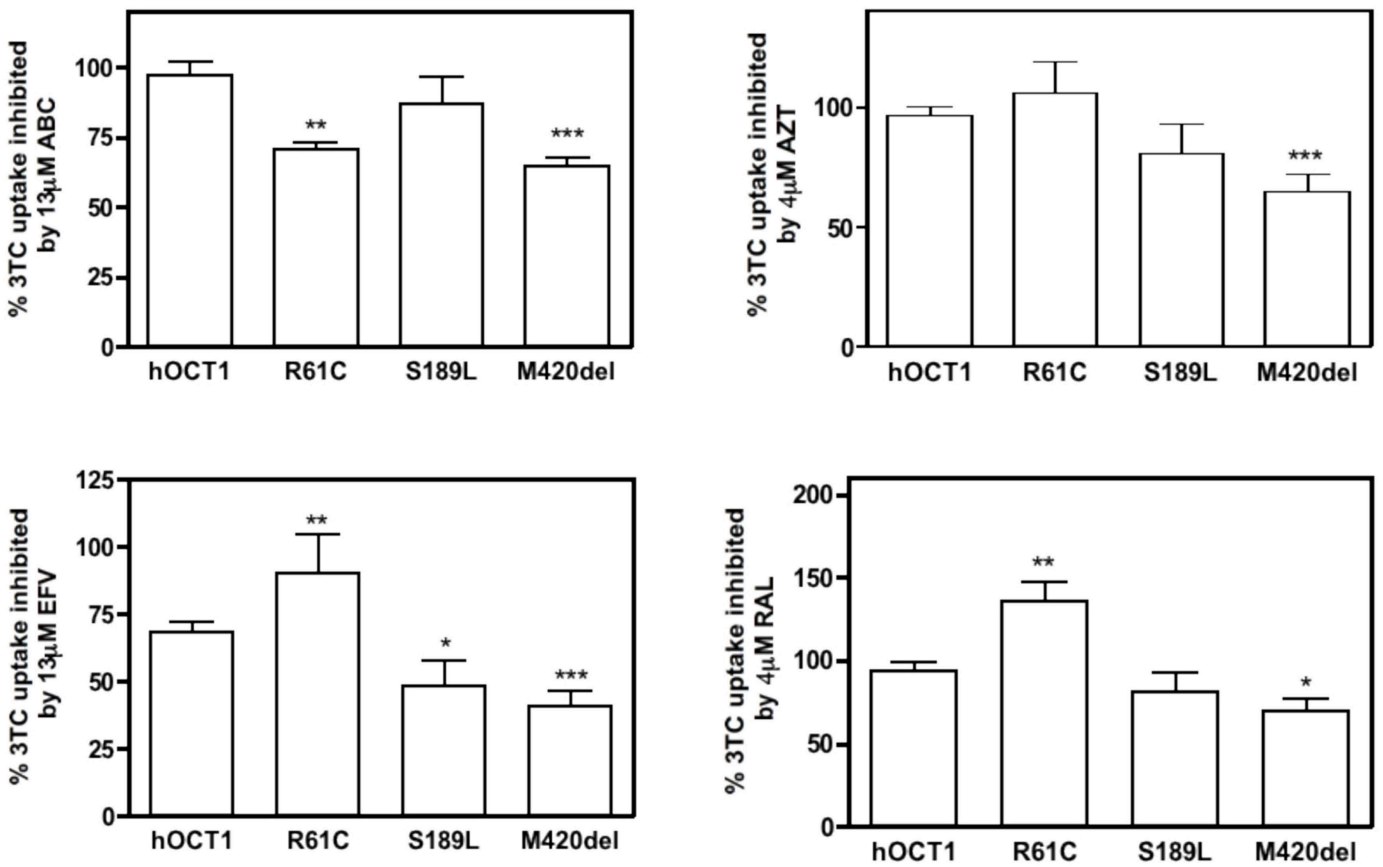
**Cis-inhibition studies of 3TC with other antiretroviral drugs in hOCT1 polymorphic variants**. 3TC (10 μM, 2 μCi) uptake was inhibited with a dose close to the *Cmax* value of other antiretroviral drugs in HEK293 stably expressing the WT transporter or one of its polymorphic variants. Graphs show the mean ± S.E.M of three to four single experiments. (^*^p < 0.05; ^**^p < 0.01; ^***^p < 0.001).

The functional impact on drug-drug interactions of polymorphic variants relevant to the clinics was studied using additional *cis-inhibition* assays. For this purpose, HEK293 cells stably expressing either the wild type hOCT1 or the selected variants were used. As mentioned above, only those variants for which *Km* and *Vmax* values could be calculated were chosen for drug-drug interaction assays. As shown in Figure 4, significant differences were observed for each assessed interaction (ABC, AZT, EFV, and RAL, each tested against 3TC uptake) among polymorphic variants. ABC, despite not being able to significantly inhibit the wild type hOCT1-mediated uptake of 3TC could significantly inhibit its uptake mediated by the polymorphic variants R61C and M420del. AZT could only significantly inhibit the uptake mediated by the polymorphic variant M420del. EFV, as mentioned before, was able to inhibit 3TC transport mediated by the wild type transporter, although it could not block R61C function. Interestingly, EFV was also more capable to inhibit 3TC uptake mediated by the S189L and M420del variants than that associated with the wild type transporter. Interestingly, RAL co-administration with 3TC did not reduce but even increased 3TC uptake mediated by the polymorphic variant R61C whereas it could slightly decrease its transport when this was mediated by the M420del polymorphic variant (Figure 4).

## Discussion

This study demonstrates that selected hOCT1 polymorphic proteins, some of them showing relatively high allelic frequency in humans, may show variable efficacy for drug uptake, in this case for 3TC translocation, but also variable interaction with co-administered antivirals known to be high affinity inhibitors of hOCT1-mediated 3TC uptake (Minuesa et al., 2009). All interacting drugs were assayed at *Cmax* concentrations. Although this may not necessarily reflect free unbound circulating drug levels, they are closer to the clinics, thereby anticipating that interactions may potentially modulate 3TC pharmacokinetics and pharmacodynamics. This issue will require further clinical pharmacological approaches which are beyond the scope of this contribution. In this particular case we focused on the molecular variants of hOCT1 and their drug-interaction properties, thereby providing novel basic knowledge to the HIV-1 infection therapeutics field. However, the clinical impact of hOCT1 genetic heterogeneity in humans has been studied for several drugs so far, being metformin a paradigm among them. The three variants here studied in more detail, R61C, S189L, and M420del, have been reported to determine decreased metformin uptake. For the two latter at least, kinetic analyses revealed that low activity was mostly accounted by decreased *Vmax* values, without significant *Km* changes (Shu et al., 2007). In this report, although *Km* values were significantly increased (nearly 50% vs. the wild type transporter) for the S189L variant when looking for 3TC transportability, it is similarly evident that the most striking alteration in 3TC uptake kinetics was associated with decreased *Vmax* values. Overall, this resulted in a dramatic loss of transport efficacy (*Vmax/Km* ratio). The possibility that these alterations would be a mere consequence of altered transporter expression levels is unlikely because kinetic changes are not of the same magnitude than the ones found for the hOCT model substrate MPP^+^. In this regard transport efficacy is not even significantly impaired when analyzing the performance of the S189L and M420del variants using MPP^+^ as substrate, which is not the case when determining 3TC uptake kinetic parameters. Moreover, as discussed below, altered drug-drug interaction properties with hOCT1 polymorphic variants are totally independent on transporter expression levels but, instead, rely upon inherent transporter translocation mechanisms. More importantly, the probable link between the occurrence of these three variants, R61C, S189L, and M420del and altered drug pharmacokinetics has also been studied in humans receiving metformin. In all cases, individuals bearing these polymorphic transporters showed increased Area Under the Curve (AUC) and *Cmax* values for this drug (Shu et al., 2008), thereby suggesting that there might be a direct link between altered function of hOCT1 proteins and *in vivo* metformin handling. Moreover, although still controversial, the occurrence of these variants has also been reported to associate with high metformin renal clearance (Tzvetkov et al., 2009). Thus, it is highly likely that similar effects could be anticipated for other hOCT1 substrates, such as 3TC. Overall, these observations are consistent with the known tissue distribution of hOCT1 in humans. hOCT1 is expressed at the sinusoidal (basolateral) membrane of hepatocytes (Nies et al., 2009) and at the apical and sub-apical domains of proximal and distal tubules in human kidney (Tzvetkov et al., 2009). It is also apically located in small intestine, and in general, is present in many other epithelia (Koepsell et al., 2007; Han et al., 2013). In summary, as briefly introduced above, hOCT1 localization in the main epithelial barriers of the body makes it a suitable candidate to modulate the pharmacokinetics of hOCT1 substrates. Not only genetic heterogeneity may apply to pharmacologically relevant epithelial barriers but also, to target cells. In this regard, hOCT1 in the sinusoidal membrane of hepatocytes can contribute to metformin effects in antidiabetic therapy, as long as the hepatocyte is by itself a cell target for treatment. It may also determine the antiviral efficacy of 3TC itself, in HBV therapy. Moreover, despite some controversy still exists about whether hOCT1 is a sorafenib transporter or not, it has recently been shown that sorafenib uptake and cytotoxicity might be affected by hOCT1 gene heterogeneity in humans (Herraez et al., 2013). Bendamustine, a drug currently used in front line treatment of Chronic Lymphocytic Leukemia (CLL) also appears to be a hOCT1 substrate and genetic heterogeneity in CLL patients appears to account for a subset of cases with altered drug sensitivity (Arimany-Nardi et al., 2015b). But besides gene heterogeneity, the abundance of the transporter itself might eventually contribute to drug action. Thus, even though particular somatic hOCT1 mutations have been recently identified in hepatocarcinomas (Herraez et al., 2013), decreased expression of this drug transporter has also been reported when compared to adjacent non-tumor tissue (Martinez-Becerra et al., 2012). In this regard, it should be noted that high variability in hOCT1 expression has also been reported in healthy livers among individuals (Nies et al., 2009). hOCT1 is also known to be expressed in most immune system cells, including a major target in AIDS therapy, CD4^+^ T cells (Minuesa et al., 2008; Jung et al., 2013). Moreover, hOCT1 expression also appears to be up-regulated when cells are stimulated by cytokines, mimicking what could be happening in the HIV-1 infection context (Minuesa et al., 2008). Overall this highlights the possibility of drug handling being determined by both, transporter abundance and genetic heterogeneity.

Although hOCT1 is a highly polymorphic transporter, which has been quite well studied for a decent amount of substrates so far, there is still little information about how genetic heterogeneity can affect drug-drug interactions. This issue might be relevant in therapeutic regimes aimed at treating HIV-1 infection and, probably other viral infectious diseases. 3TC-based antiretroviral regimens have been widely used in HIV-1 therapies. The drug was first co-formulated with AZT (Combivir®), and subsequently with ABC (Kivexa®). Fix-dose combinations of 3TC have been also manufactured with AZT+ABC (Trizivir®), and more recently with ABC+dolutegravir (Triumeg®). In 2009, the FDA granted approval for generic formulations of 3TC. In this regard, the fact that R61C and M420del variants showed a more variable profile of drug-drug interactions when compared to the wild-type protein highlights the importance that these polymorphisms can have in drug bioavailability and action when administered in drug combinations.

A major issue in the field would be how to predict this type of interactions based upon structure-function knowledge of the hOCT1 protein. This could be useful for new drug development and could also be eventually extended to other members of the *SLC22* gene family, sharing high homology with hOCT1. Nevertheless, structural knowledge is still poor. Moreover, an additional element of complexity is added to this problem, this is the fact that the fully functional transporter is built up as an oligomer and the functional impact of the variants on the monomers crosstalk and, in particular of those polymorphisms located in the first extracellular loop of the protein, are very difficult to predict.

In summary, this study provides novel insights on how 3TC interacts with hOCT1 polymorphic proteins. It also addresses the very interesting question of how genetic heterogeneity would determine variable drug-drug interactions, an issue of particular relevance in AIDS treatment. It should be noticed that some polymorphic variants showing great functional complexity, such as M420del, are present in humans with high allelic frequency and thus, it is likely to play a major impact in drug pharmacokinetics under combined therapies. Overall, this study highlights the need for establishing functional assays aiming at anticipating drug-drug interactions at the early stages of drug development.

## Author contributions

Participated in research design: CA, GM, HK, JM, and MP. Conducted experiments: CA, GM, TK, and IE. Contributed new reagents or analytic tools: HK. Performed data analysis: CA, GM, HK, JM, and MP. Wrote or contributed to the writing of the manuscript: CA, GM, HK, JM, and MP.

## Funding

This study was supported by research funding from Spanish Secretariat of Research (SAF2011-23660 and SAF2014-52067-R to MP; SAF2013-49042-R to JM) and FEDER (European Union). This study was also supported by Deutsche Forschungsgemeinschaft Grant KO 872/6-1 to HK. CA was recipient of predoctoral fellowships FPI from Ministerio de Ciencia e Innovación.

### Conflict of interest statement

The authors declare that the research was conducted in the absence of any commercial or financial relationships that could be construed as a potential conflict of interest.

## Abbreviations

hOCT: Human organic cation transporter
3TC: lamivudine
WT: wild type
TMD: transmembrane domain
HIV: Human Immunodeficiency Virus
HBV: Hepatitis B virus
ABC: abacavir
AZT: azidothymidine
AIDS: acquired immune deficiency syndrome
MDMs: Monocyte-Derived Macrophages
MDDCs: Monocyte-Derived Dendritic Cells
PBMCs: Peripheral Blood Mononuclear Cells
MPP^+^: N-Methyl-4-phenyl pyridinium iodide
FTC: emtricitabine
TDF: tenofovir diproxil fumarate
EFV: efavirenz
RAL: raltegravir
HEK: Human embrionic kidney
NRTI-: Nucleoside Reverse Transcriptase Inhibitors
NNRTI: Non-Nucleoside Reverse Transcriptase Inhibitor.
