# Supplementary material for: Role of Human Organic Cation Transporter 1 (hOCT1) Polymorphisms in Lamivudine (3TC) Uptake and Drug-Drug Interactions
Source: Front Pharmacol. 2016 Jun 24;7:175. doi: 10.3389/fphar.2016.00175 (PMC4919327; doi:10.3389/fphar.2016.00175)

Supplementary figure 1. Uptake rates of 10 µM [^{3}H]3TC and 1 µM [^{3}H]MPP$^+$ by the wild type hOCT1 and its polymorphic variants. Measured in in traniently transfected HeLa cells. The figure shows the mean +/- S.E.M of three to five single measurements (p<0.05, *; p<0.001,***)

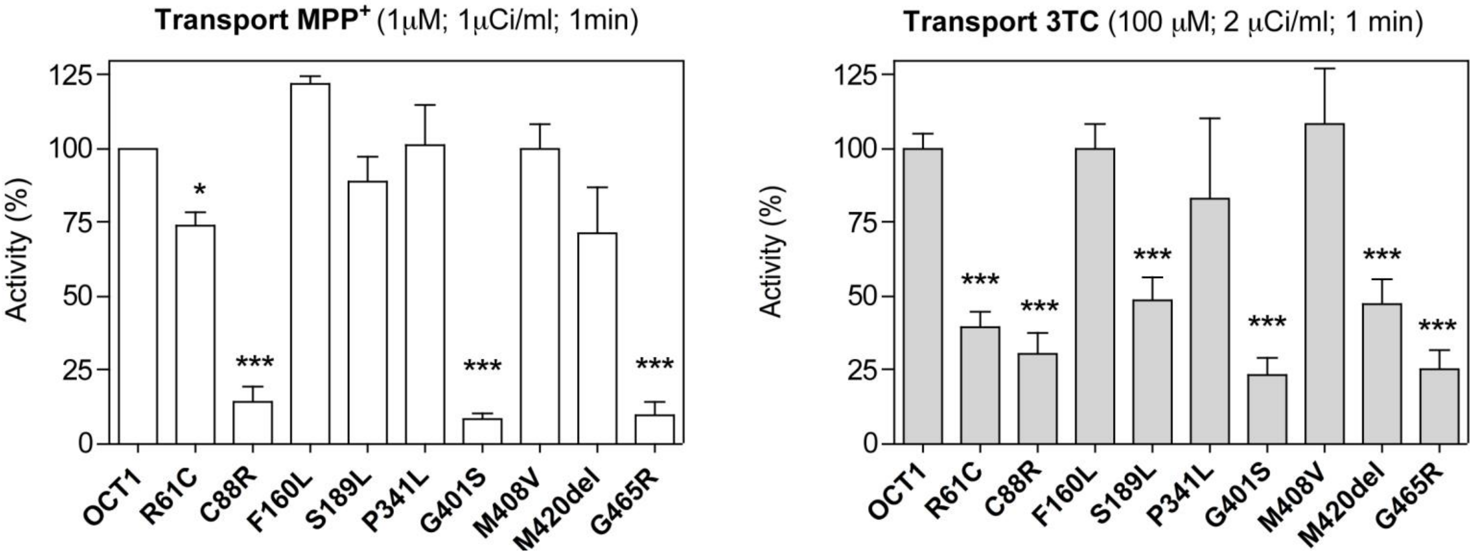

Supplementary figure 2. MPP+ mediated uptake in HEK293 cells at 1s. Measured in stably transfected HEK293 cells. The figure shows the mean +/- S.E.M of three to five single measurements.

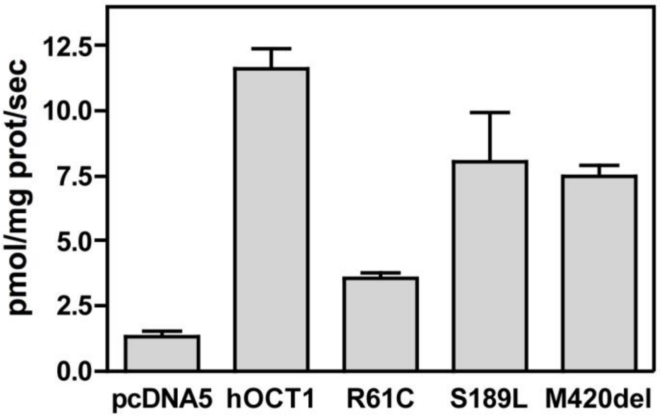

Supplement: Supplementary file 1 [file Image1.PDF]
